# Radioprotective Effects from Propolis: A Review

**DOI:** 10.3390/molecules28155842

**Published:** 2023-08-03

**Authors:** Blanca Ibáñez, Ana Melero, Alegría Montoro, Nadia San Onofre, Jose M. Soriano

**Affiliations:** 1 Food & Health Laboratory, Institute of Materials Science, University of Valencia, 46980 Paterna, Spain; 2 Department of Pharmacy and Pharmaceutical Technology and Parasitology, Faculty of Pharmacy, University of Valencia, 46100 Burjassot, Spain; 3 Service of Radiological Protection, Clinical Area of Medical Image, University and Polytechnic La Fe Hospital, 46026 Valencia, Spain; 4 Biomedical Imaging Research Group GIBI230, Health Research Institute (IISLaFe), University and Polytechnic La Fe Hospital, 46026 Valencia, Spain; 5 Department of Community Nursing, Preventive Medicine and Public Health and History of Science, University of Alicante, 03690 Alicante, Spain; 6 Joint Research Unit on Endocrinology, Nutrition and Clinical Dietetics, University of Valencia-Health Research Institute La Fe, 46026 Valencia, Spain

**Keywords:** radioprotection, propolis, radiation

## Abstract

Propolis is a natural bee-produced substance with antimicrobial, anti-inflammatory, and wound-healing properties, containing some components from the leaves, buds and resins of plants. It has been used for centuries for various health benefits. In this manuscript, our group reviewed the radioprotective effect of propolis using PubMed and Embase, and our review was conducted according to the PRISMA statement. Finally, 27 articles were included in this review, which includes the radioprotective effect of propolis from cell-based studies (*n* = 8), animal models (*n* = 14), and human trials (*n* = 5). Results reflected that the dosage forms of propolis extracted in the scientific literature were ethanolic extracts of propolis, a water-soluble derivate of propolis, or capsules. The efficacy of the radioprotective properties from propolis is extracted from the bibliography, as several compounds of this resinous mixture individually or synergistically are possible candidates that have the radioprotective effect. In fact, studies prior to 2011 lacked a comprehensive characterization of propolis due to the variability in active compounds among different batches of propolis and were limited to analytical techniques. Furthermore, in this manuscript, we have selected studies to include primarily propolis types from Brazil, Croatia, Egypt, European countries, and those commercialized in Spain. They all contained ethanolic extract of propolis (EEP) and were influenced by different dosage forms. EEP showed a significant presence of lipophilic bioactive compounds like flavones, flavonols, and flavanones.

## 1. Introduction

Propolis is a resinous mixture that bees collect from various botanical sources, including buds, flowers, and plant exudates. Bees modify the collected resins by adding their salivary secretions, wax, and pollen, resulting in the creation of propolis. The collection of resins by bees typically takes place during the hottest hours of the day when the resins are more malleable and easier to gather [1]. Due to its balsamic and resinous nature, propolis has a highly sticky consistency. It encompasses a diverse array of components, including terpenes, polysaccharides, aromatic acids, polyphenols, esters of phenolic acids, vitamins, and amino acids [2]. However, propolis can vary in composition and effectiveness depending on its botanical sources and geographical origin [3]. Propolis has long been associated with various medicinal properties and has been utilized for its potential health benefits. Some of the attributed medicinal properties of propolis include binder, immunomodulator, antimicrobial, antifungal, anti-inflammatory, hepatoprotective, antioxidant, anti-hemorrhagic, anti-parasitic, anti-tumor, anti-edema, stimulating epithelial regeneration, cholesterol reducer, revitalizing, detoxifying and tonic [4,5,6]. The objective of this review was to assess the radioprotective effect of propolis in different experimental settings, including studies involving cells, animals, and humans.

## 2. Results and Discussion

Table 1, Table 2 and Table 3 show the radioprotective effect of the propolis including cell-based studies, animal models, and human trials, respectively.

For cell-based studies, six out of eight articles [7,8,9,10,11,12] reflected the use of ionizing radiation in their research, while the other two referenced the application of UV radiation, including UV-B [13] and UV-A [14]. Furthermore, six out of eight articles [7,8,9,10,11,13] indicated the use of ethanolic extract of propolis (EEP) compared to an aqueous solution of propolis [12] and wax from *Tetragonula* spp. bees [14]. These species include *T. biroi*, *T. fusco balteata*, *T. laeviceps* and *T. sapiens* [34], which produce more propolis than other bee species [35]. On the other hand, ^60^Co γ-irradiation was mainly used in five out of six articles [7,8,9,11,12], with only one unique application of X-rays in ionizing radiation [10], with the radiation range from 1 to 6 Gy.

**Figure 1:**
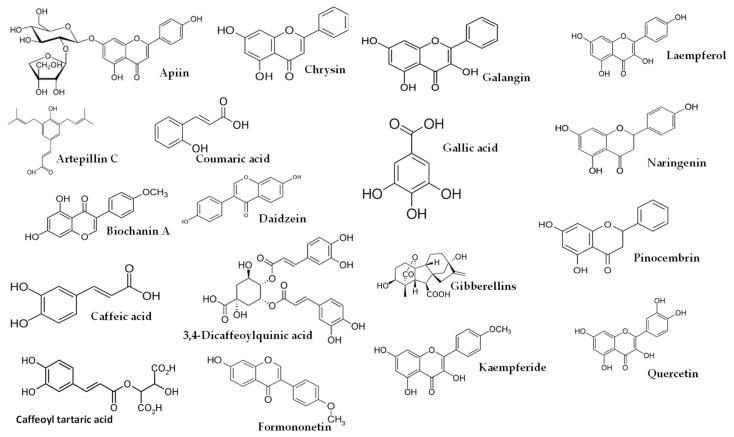
Some of compounds detected from Brazilian, Croatian and Egyptian propolis.

**Figure 2:**
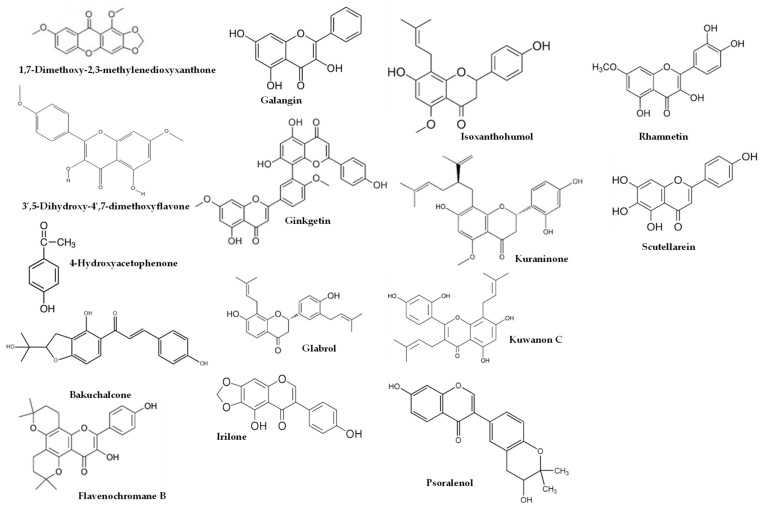
Some of compounds detected from propolis commercialized in Spain and European propolis.

**Figure 3:**
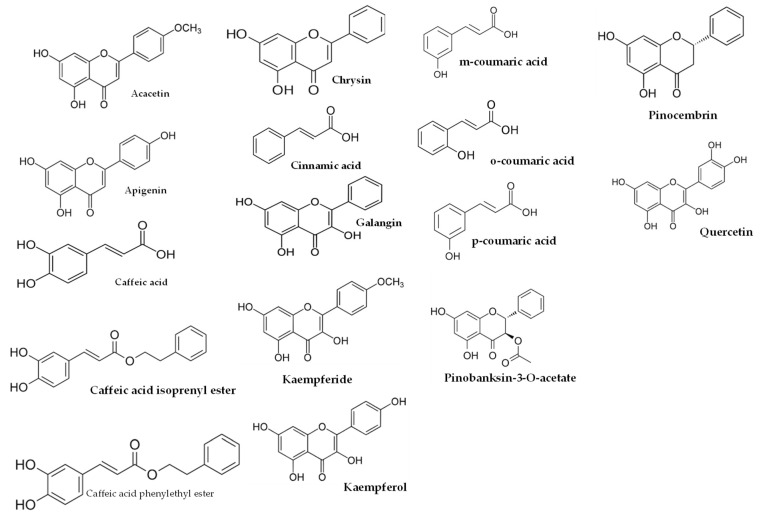
Some of compounds of propolis wax from *Tetragonula* sp. bees.

For non-human animals, male rats were used more frequently, primarily Wistar rats [21,22,23,24,25,27,28], which were followed by Sprague–Dawley rats [26]. In addition, CBA mice [15,16,17,18,19,20] were also utilized. Various types of propolis were employed, including EEP [19,20,21], water-soluble derivative of propolis (WSDP) [15,16,17,18,28], and hydroalcoholic extract of red propolis (HERP) [27]. In terms of radiation, X-rays were utilized in one study at a dose rate of 15 Gy [21], while γ-irradiation was mainly obtained using ^60^Co [15,16,17,18,19,20,23,24,25,26], which was followed by ^137^Cs [22,28]. The radiation range for both types of radiation was from 1 to 15 Gy, with the latter applied to the entire cranium. Additionally, non-ionizing radiation in the form of UV-B was only detected in one article [27], where it was applied at a dosage of 1.6 J/cm^2^ for 60 min per day over a period of 6 days. For humans, several studies were conducted involving male participants [29,30], female participants [32], or both genders [31,33], all aged 18 years or older. The dosage forms of propolis used in these studies were EEP [29], WSDP [30,31], or capsules [32]. In three out of five articles [29,30,31], the use of ^60^Co γ-irradiation was predominant, with a radiation range of 2 Gy [31] to 4 Gy [29,30]. The human subjects in these studies were individuals with head and neck malignancies [31], patients diagnosed with oral cavity or oropharynx cancer [33], and breast cancer patients [32]. The studies observed various improvements in several parameters, such as the prevention and healing of radiotherapy-induced mucositis [31], protection against DNA damage induced by ionizing radiation in leukocytes of breast cancer patients [32], and propolis treatment resulting in a reduction in TNF-α and IL-1β levels as well as a decrease in the incidence of oral candidiasis episodes [33]. These findings suggest that propolis could serve as a beneficial complementary option for the prevention and treatment of the primary acute oral toxicities associated with radiotherapy [33]. In the included studies for this manuscript, it is clear that the studies prior to 2011 did not have the complete characterization of the propolis extracts. This could be attributed to two reasons. Firstly, it was probably due to the variability of bioactive compounds among different batches of propolis used, and secondly, it could be due to the limited development of analytical techniques, particularly chromatography coupled with mass spectrometry, in analytical laboratories at that time. The only study [16] in 2005 provided a partial characterization of the propolis used. It mainly focused on caffeic acid, naringenin, chrysin, pinocembrin, galangin, and total polyphenols. In 2011, our group [9] emphasized the importance of comprehensively characterizing the extracts used in studies investigating the biological activities of propolis. This advancement allowed subsequent articles to include detailed analyses of the propolis composition used in radioprotection studies within a few months [10] and in the following years [13,14,25,27,33]. Table 4 presents the broad composition of these preparations or extracts, emphasizing the significance of their composition. Furthermore, some compounds of this table are reflected in Figure 1, Figure 2 and Figure 3.

As shown in Table 4, higher dosages had an impact on the composition of the studies using EEP, resulting in a higher proportion of bioactive lipophilic compounds such as flavones, flavonols, and flavanones. Furthermore, this manuscript covered several propolis origins, including Brazilian [13,16,27,33], Croatian [13,16], Egyptian [25], European [10], and Iranian [24] propolis, as well as propolis commercialized in Spain [9]. It is worth noting that green or red propolis originated from Brazil. In the studies conducted by Park et al. [39,40] and Shimizu et al. [41], various constituents of propolis were identified, including quercetin, apigenin, kaempferol, acacetin, melliferone, moronic acid, anwuweizonic acid, betulonic acid, 4-hydroxy-3-methoxy-propiophenone, 4-hydroxy-3-methoxybenzaldehyde, 3-(3,4-dimethoxyphenyl)-2-propenal, and acetoxitremetone. However, major compounds such as artepillin C and chrysin were not found in their analyses. In addition, Santos et al. [11] suggested that propolis of Brazilian origin was reported to lack caffeic acid phenethyl ester (CAPE) in its chemical composition. Until 2016, the commercially available propolis was primarily extracted Brazilian green propolis. However, a new type of propolis emerged and was widely known as Brazilian Organic Propolis (BOP) [33,34]. BOP is characterized by its mild flavor and is produced under organic conditions in conservation areas. It is noteworthy that BOP is free from heavy metals and pesticides, making it a desirable option. BOP has demonstrated interesting bioactive properties. For instance, BOP type 4 has significant antioxidant activity, which is mainly due to its high content of Artepillin C. On the other hand, BOP type 6 has shown remarkable anti-inflammatory activity by reducing NF-kB activation and TNF-α release [33]. These properties are particularly relevant, since oral mucositis pathogenesis is closely related to inflammatory pathways and the production of reactive oxygen species. As a result, both types of BOP have become subjects of investigation for their potential in addressing oral mucositis [42]. Another type of propolis is Brazilian red propolis, which contains significant amounts of flavonoids, such as pinocembrin, formononetin, and isoliquiritigenin [43]. Additionally, HERP (hydroalcoholic extract of red propolis) exhibits antioxidant properties [44,45] and demonstrates anti-inflammatory effects [46]. Moreover, HERP is hypothesized to have the potential to protect the skin from damage caused by UV-B radiation, which is attributed to the presence of benzophenone in HERP, a compound commonly used in the formulation of organic sunscreens. These unique properties make HERP a compelling subject of interest in research and potential applications.

On the other hand, the mechanisms of action of propolis are grouped in four properties [9,47]. (i) The first is antioxidant activity, as propolis is rich in various bioactive compounds, including flavonoids, phenolic acids, and polyphenols, which have strong antioxidant properties. When exposed to ionizing radiation, the body generates free radicals that can cause cellular damage. Propolis’ antioxidants can neutralize these free radicals, reducing oxidative stress and preventing further damage to cells. (ii) The second mechanism is DNA repair stimulation. In fact, radiation can cause breaks and lesions in DNA strands, leading to mutations and cell death. Propolis may enhance the body’s natural DNA repair mechanisms, helping to fix some of the radiation-induced DNA damage and increasing the chances of cell survival. (iii) The third mechanism is anti-inflammatory effects. Propolis exhibits anti-inflammatory properties, which can help mitigate inflammation caused by radiation exposure. Inflammation contributes to tissue damage and can exacerbate the harmful effects of radiation and (iv) immune system modulation. Propolis has been shown to modulate the immune system, potentially enhancing the body’s defense against radiation-induced damage. By supporting the immune system, propolis may aid in the recovery of damaged tissues.

## 3. Materials and Methods

**Figure 4:**
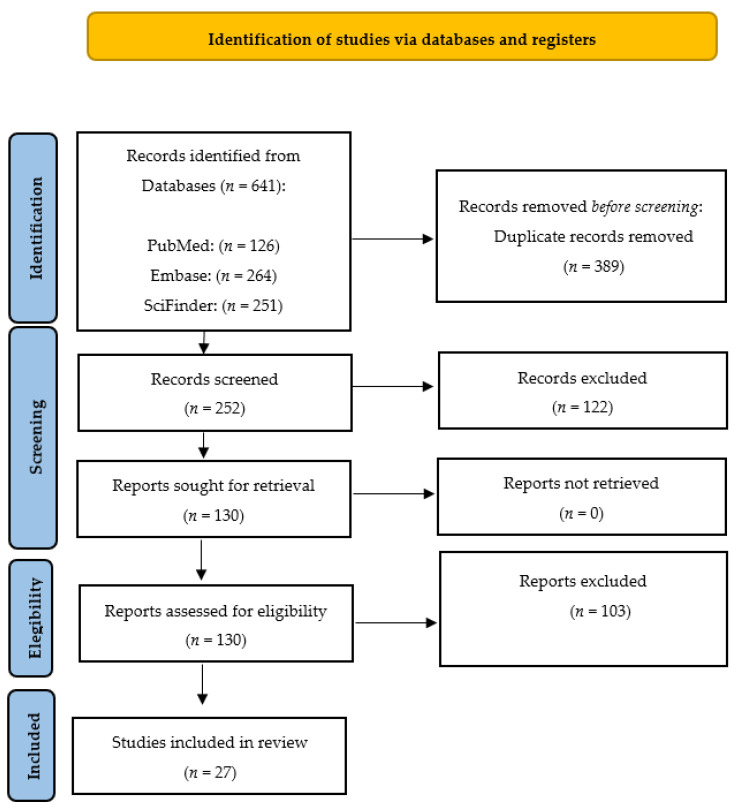
PRISMA (Preferred Reporting Items for Systematic Reviews and Meta-Analyses) 2020 flow diagram representing the searching and selection process for this review [48].

We conducted a systematic review following the guidelines outlined in the Preferred Reporting Items for Systematic Reviews and Meta-Analyses (PRISMA) statement [48] (Figure 4). Our search was performed using the PubMed, SciFinder and Embase databases. In PubMed, we used the medical subject headings (MeSH) terms ‘propolis’, ‘radioprotection’, and ‘radiation’. In Embase, we searched for the terms ‘vitamin’, ‘radioprotection’, and ‘radiation’. We employed Boolean operators (AND, OR) to combine the keywords in our search. The search was limited to literature published in the English language over the last 50 years and updated to 1 June 2023. The inclusion criteria were articles written in English from original papers, review papers, theses and experimental procedures. The exclusion criteria for this study were manuscripts with unrelated abstracts, books, letters, conference literature, case reports, editorials, and pilot studies. The full-text articles of all the shortlisted studies were then thoroughly examined to determine their eligibility. Two teams of paired reviewers, each with expertise in medical and health assessment and training in research methodology, conducted the screening process independently. The three sections of the articles, namely titles, abstracts, and full texts (if eligible), were meticulously reviewed. Additionally, the reviewers assessed the applicability of the studies and collected relevant data for the reviews. The teams worked separately to ensure a comprehensive and unbiased assessment of the articles under consideration.

## Figures and Tables

**Table 1:** Radioprotective effect of propolis in cell-based studies.

Type of Propolis Preparation (Concentration)	Radiation Type and Dose	Studied Sample	Main Outcomes	Refs.
EEP (3–33 µg/mL)	^60^Co γ-irradiation (1 Gy)	CHO-K1 cells	Decrease tendency in the quantity of radio-induced damage on cells that had been pre-treated with EEP	[7]
EEP (pretreated cells during 15 and 30 min with concentrations of 100, 200 and 300 μg/mL)	^60^Co γ-irradiation (3 Gy with 600 s of total time of exposure to radiation)	Fibroblast cells	Reduction in γ-ray-induced DNA damage in cells that were treated with EEP	[8]
EEP (20, 40, 120, 250, 500, 750, 1000, and 2000 μg/mL)	^60^Co γ-irradiation (2 Gy with 600 s of total time of exposure to radiation)	Human lymphocytes	Decrease in the frequency of chromosome aberrations in samples treated with EEP. The protection against the formation of dicentrics, a specific type of chromosome aberration, exhibited a concentration-dependent pattern, with the maximum protection observed at a concentration of 120 μg/mL of EEP. The relationship between the observed frequency of dicentrics and EEP concentration followed a negative exponential function, suggesting that approximately 44% of dicentrics could be effectively protected against at the maximum concentration of EEP	[9]
EEP (0–250 mg/mL; 1, 4, 24 h)	X-rays (0–6 Gy, single dose)	HNSCC cell lines (FaDu, UT-SCC15, UT-SCC45), fibroblasts (HSF2) and keratinocytes (HaCaT)	Reduction in cell growth and clonogenic survival, following a time- and concentration-dependent pattern. Additionally, propolis induced apoptosis, as evidenced by Caspase 3 cleavage. Furthermore, propolis treatment led to an increased phosphorylation of extracellular signal-regulated kinase 1/2 (ERK1/2), protein kinase B/Akt1 (Akt1), and focal adhesion kinase (FAK)	[10]
EEP (50, 100, and 200 μg/mL of EEP and 30, 50, and 100 μg/mL of EEP for cell viability and clonogenic assays, respectively)	^60^Co γ-irradiation (dose rate of 2.82 Gy/min with a 90% attenuator, at doses of 1, 2, 4 and 6 Gy)	CHO-K1 cells	Demonstrate, at 30 μg/mL, a radio-protective effect by reducing radiation-induced DNA damage in irradiated CHO-K1 cells. Moreover, at a concentration of 50 μg/mL, propolis exhibited a significant proliferative effect when combined with radiation, resulting in a decrease in the percentage of necrotic cells. Additionally, the concentration of 50 μg/mL of propolis showed a significant stimulating effect on cell proliferation	[11]
*Tetragona clavipes* propolis extracts (aqueous solution at concentrations of 0.5, 1, 5 and 10%)	^60^Co γ-irradiation (2 and 5 Gy)	Human Peripheral Blood Mononuclear Cells (PBMCs)	Increase in cell viability, particularly in the 5% and 10% concentrations of the extract, when incubated in culture, even after exposure to a radiation dose of 5 Gy	[12]
EEP of Brazilian Green Propolis	5 mJ/cm^2^ of UV-B light for 7 s and after 6 h from UVB irradiation	Human skin derived-immortal keratinocyte cell line, HaCaT cells	In weakly UVB-exposed cells, the application of propolis was found to effectively suppress TJ (tight junction) permeability, reactive nitrogen species (RNS) production, and the nitration level of CLDN1	[13]
Propolis wax from *Tetragonula* sp. bees	4 J/cm^2^ of UV A light	Human Embryonic Kidney 293 T and fibroblast cells lines	Provide protection to cells against the formation of free radicals induced by UV radiation. This protection was achieved by maintaining the cell proliferation rate, reducing the production of free radicals following UV exposure, and decreasing the number of cell deaths	[14]

EEP: Ethanolic extract of propolis.

**Table 2:** Studies of radioprotective effect of propolis in non-human animals.

Type of Propolis Preparation (Concentration)	Radiation Type and Dose	Studied Sample	Main Outcomes	Ref.
WSDP (per OS via gastral tube, daily for 20 and 40 days and the daily dose contained 50 mg/kg bw)	^60^Co γ-irradiation (4–9 Gy)	3-month-old CBA male mice	In mice treated with WSDP, both the number of endogenous and exogenous colony-forming units (CFUs) in the spleen was found to be higher compared to the control group. Notably, when using the exogenous CFUs assay, it was observed that CFUs derived from the spleen of WSDP-treated mice exhibited greater resistance to irradiation compared to CFUs from the spleen of normal mice	[15]
WSDP of Croatian and Brazilian propolis (50 or 150 mg/kg)	^60^Co γ-irradiation (9 Gy)	3-month-old CBA female mice	WSDP has been shown to possess radioprotective, stimulative, and regenerative properties on hematopoiesis. These findings suggest that WSDP could have clinical potential in the treatment of various cytopenias induced by radiation and/or chemotherapy	[16]
WSDP and EEP (100 mg kg^−1^ bw i.p. for 3 consecutive days before (pretreatment) or after irradiation (therapy))	^60^Co γ-irradiation (9 Gy)	3-month-old CBA male mice	Their use has been observed to provide protection against hematopoietic death, which refers to mortality occurring within 30 days after irradiation	[17]
WSDP (100 mg kg^−1^ bw i.p. for 3 consecutive days before (pretreatment) or after irradiation (therapy))	^60^Co γ-irradiation (4 and 9 Gy)	2-month-old CBA male mice	WSDP given to mice before irradiation protected mice from lethal effects of whole-body irradiation and diminished primary DNA damage in their white blood cells	[18]
EEP (100 mg kg^−1^ bw i.p. for 3 consecutive days before (pretreatment) or after irradiation (therapy))	^60^Co γ-irradiation (9 Gy)	3-month-old CBA male mice	EEP, before irradiation, protected white blood cells of mice from lethal effects of irradiation and diminished primary DNA damage	[19]
EEP (100 mg kg^−1^ bw i.p. for 3 consecutive days before (pretreatment) or after irradiation (therapy))	^60^Co γ-irradiation (4 Gy)	3-month-old CBA male mice	Mice that received pretreatment with EEP demonstrated reduced sensitivity to irradiation. On the other hand, mice that received post-irradiation therapy with EEP showed a slight increase in total leukocyte count compared to the irradiated negative control, although the increase was not statistically significant. Notably, EEP exhibited a protective effect against primary DNA damage in leukocytes of mice	[20]
EEP (injections of 100 or 200 mg/kg i.p. of EEP)	X-rays (a dose rate of 15 Gy in 9 min and 39 s)	7–11-week-old Wistar male rats	Reduce and delay radiation-induced mucositis in this animal model	[21]
WSDP (5 mL/kg of the 13% WSDP solution (corresponding to 0.65 g dry extract/kg))	^137^Cs γ-irradiation (single radiation dose level of 2, 3 or 6 Gray (Gy) being the radiation dose rate was 0.48 Gy/min)	Wistar male rats	Reduce the number of gastric lesions as well as the plasma level of malondialdehyde (MDA)	[22]
400 mg/kg propolis injections before irradiation	^60^Co γ-irradiation (15 Gy, on the whole cranium for 7 min and 39 s)	Wistar albino male rats	Protect propolis of effects on salivary gland function in animal models whilst it did not prevent radiation-induced histologic changes in tissues	[23]
Iranian propolis	^60^Co γ-irradiation (5 Gy of irradiation for 7 min and 39 s)	7–11-week-old Wistar male rats	Reduce and delay radiation-induced mucositis in animal models	[24]
Propolis was freshly prepared and administered to animals orally at a dose of 90 mg/kg	^60^Co γ-irradiation (1 Gy every day up to 5 Gy total doses)	Albino male rats	Propolis can be more effective than honey in the protection against oxidative damage induced by ionizing radiation	[25]
30% in propolis, 40% in caffeic acid phenethyl ester (CAPE), 20% in Nigella sativa oil (NSO) and 50% thymoquinone (TQ) administered by either orogastric tube or i.p. injection	^60^Co γ-irradiation (5 Gy)	Sprague–Dawley male rats	Propolis, CAPE, NSO, and TQ could prevent cataractogenesis in ionizing radiation-induced cataracts in the lenses of rats, wherein propolis and NSO were found to be more potent	[26]
Hydroalcoholic Extract of Red Propolis (HERP)	1.6 J/cm^2^ of UV B light	Adult Wistar male rats	HERP might protect the skin against tissue damages caused by UVB radiation	[27]
WSDP (3 days before exposure, rats were given WSDP orally and treatment continued for 2 more days)	^137^Cs γ-irradiation (8 Gy)	Wistar male rats	Diminish apoptotic indicators and oxidative stress parameters	[28]

WSDP: Water-soluble derivate of propolis.

**Table 3:** Studies of radioprotective effect of propolis in humans.

Type of Propolis Preparation (Concentration)	Radiation Type and Dose	Studied Sample	Main Outcomes	Reference
EEP (pretreated blood samples with for 30 and 120 min with 100 µg mL^−1^ of EEP)	^60^Co γ-irradiation (4 Gy)	One healthy male donor (age 35 years, non-smoker)	Diminish the levels of primary and more complex cytogenetic DNA damage in γ-irradiated white blood cells	[29]
WSDP (pretreated blood samples with for 30 and 120 min with 100 µg mL^−1^ of WSDP)	^60^Co γ-irradiation (4 Gy)	One healthy male donor (age 35 years, non-smoker)	Reduce the number of necrotic cells and to diminish the levels of primary and more complex cytogenetic DNA damage in white blood cells	[30]
15 mL of WSDP (3%) and then to swallow 3 times/day for 5 weeks simultaneously with radiotherapy protocol from the first session	^60^Co γ-irradiation (2 Gy/day, 5 times a week up to total dose of 50 to 70 Gy)	20 patients involved with head and neck malignancies including 14 (70%) male and 6 (30%) female	Prevent and heals radiotherapy induced mucositis	[31]
Propolis capsules (400 mg, 3 times daily) for 10 consecutive days before radiotherapy, during the course of radiation treatment and 10 days after completing the radiotherapy	50 Gy over a period of 25 days in a daily fraction of 2 Gy delivered five times a week	Three groups: (i) healthy females, (ii) females with breast cancer who received chemotherapy followed by radiation therapy only and (iii) females with breast cancer who received chemotherapy followed by radiation therapy plus propolis supplements (age 35 years, non-smoker)	The supplementation of propolis in conjunction with radiotherapy treatment has demonstrated a significant protective effect against DNA damage induced by ionizing radiation in leukocytes of breast cancer patients. Additionally, propolis supplementation has been found to inhibit the overexpression of RRM2, which is a protein associated with cancer progression. Moreover, propolis exhibits beneficial effects on the antioxidant capacity of the serum and improves the absorption of iron in the digestive system as well as enhances the efficiency of hemoglobin regeneration	[32]
An aqueous suspension containing 0.8% (*w*/*v*) of the BOP mix (50% (*w*/*w*) of BOP type 4 and 50% (*w*/*w*) of BOP type 6)	A dose at least 40 Gy either adjuvant to surgery, exclusively or associated with chemotherapy	Participants had to be aged 18 years or older, diagnosed with oral cavity or oropharynx cancer	Topic use of BOP reduced TNF-α and IL-1β levels, oral candidiasis episodes, and seems to be a useful complementary option for the prevention and treatment of the main acute oral toxicities of radiotherapy	[33]

BOP: Brazilian Organic Propolis; EEP: ethanolic extract of propolis; RRM2: ribonucleotide reductase M2 subunit.

**Table 4:** Type of propolis and main compounds of selected propolis.

Type of Propolis	Main Compounds	Ref.
Croatian propolis	Contain m/V total polyphenols 14.78% (caffeic acid 2.02%, naringenin 2.41%; chrysin 2.45%, pinocembrin 3.06%, galangin 2.12%)	[16]
Brazilian propolis	Contain m/V total polyphenols 15.79% (caffeic acid 2.2%, naringenin 0.54%; chrysin 3.38%, pinocembrin 5.44%, galangin 3.07%)	[16]
Egyptian propolis	Total phenol (113.7–121.6 mg GAE/g propolis) Total flavonoid (118.3–124.5 mg CEQ/g propolis)	[25]
Brazilian red propolis	Biochanin A Daidzein Formononetin	[27]
EEP of Brazilian Green Propolis	Apiin (which is a glycoside of apigenin) Chrysin Kaempferide Laempferol Quercetin	[13]
Brazilian Organic Propolis	Caffeoyl tartaric acid Coumaric acid Caffeic acid Gallic acid 3,4-Dicaffeoylquinic acid Quercetin Gibberellins Artepillin C	[33]
Propolis commercialized in Spain	Acacetin Apigenin Caffeic acid CAPE Chrysin Cinnamic acid Galangin Kaempferide Kaempferol *m*-coumaric acid *o*-coumaric acid *p*-coumaric acid Quercetin	[9]
European propolis	Apigenin Caffeic acid Caffeic acid isoprenyl ester Caffeic acid isoprenyl ester (isomer) CAPE Kaempferide *p*-coumaric acid Pinobanksin-3-*O*-acetate Pinobanksin-3-*O*-butyrate or isobutyrate Pinobanksin-3-*O*-pentanoate or 2-methylbutyrate Pinobanksin-3-*O*-propionate Pinocembrin Quercitin	[10]
Propolis wax from Tetragonula sp. bees	1,7-Dimethoxy-2,3-methylenedioxyxanthone 3,4′,5-Trihydroxy-7-methoxy-8-isopentenyl flavone 3,5-Dihydroxy-3, 4′,7-trimethoxyflavone 3′,5-Dihydroxy-4′,7-dimethoxyflavone 4-Hydroxyacetophenone 4′-*O*-Methylbrazilin 5,7,4′-Trihydroxy-3′,8-diprenylflavone Bakuchalcone Flavenochromane B Galangin Ginkgetin Glabrol Irilone Isoxanthohumol Kuraninone Kushenol A-C, E, F, I, N, S, U, W and X Kuwanon C and E Methyl kushenol C Moracin H Psoralenol Rhamnetin Scutellarein Sophoradichromane B Sophoradichromane D	[14]
Thai propolis	19 compounds belonging to flavonoids and phenolic esters and two new compounds as are (7″*S*)-8-[1-(4′-hydroxy-3′-methoxyphenyl)prop-2-en-1-yl]- (2*S*)-pinocembrin and (*E*)-cinnamyl-(*E*)-cinnamylidenate from methanolic extract of Thai propolis	[36]
Thai propolis	27-Hydroxyisomangiferolic acid Ambolic acid Anacardic acid Cardanols Cardols Cycloartenol Isomangiferolic acid Mangiferolic acid	[37]
Thai propolis (Chanthaburi and Chiang Mai propolis)	γ- and α-mangostins and five prenylated flavanones	[38]

CAPE: Caffeic acid phenylethyl ester.

## Data Availability

Not applicable.
